# Cerebral localization of the mind and higher functions The beginnings

**DOI:** 10.1590/1980-57642018dn12-030014

**Published:** 2018

**Authors:** Eliasz Engelhardt

**Affiliations:** 1 Cognitive and Behavioral Neurology Unit, INDC - CDA-IPUB - UFRJ. Rio de Janeiro RJ - Brazil.

**Keywords:** cardiocentric, cephalocentric, brain ventricles, animal spirit, higher functions, cardiocêntrico, cefalocêntrico, ventrículos cerebrais, espírito animal, funções superiores

## Abstract

The debates about the mind and its higher functions, and attempts to locate them in the body, have represented a subject of interest of innumerable sages since ancient times. The doubt concerning the part of the body that housed these functions, the heart (cardiocentric doctrine) or the brain (cephalocentric doctrine), drove the search. The Egyptians, millennia ago, held a cardiocentric view. A very long time later, ancient Greek scholars took up the theme anew, but remained undecided between the heart and the brain, a controversy that lasted for centuries. The cephalocentric view prevailed, and a new inquiry ensued about the location of these functions within the brain, the ventricles or the nervous tissue, which also continued for centuries. The latter localization, although initially inaccurate, gained traction. However, it represented only a beginning, as further studies in the centuries that followed revealed more precise definitions and localizations of the higher mental functions.

## Resumo

Os debates sobre a mente e suas funções superiores, e as tentativas de estabelecer suas localizações no corpo, foram objeto de interesse de inúmeros sábios desde os tempos mais remotos. A dúvida quanto à parte do corpo que abrigava tais funções, o coração (doutrina cardiocêntrica) ou o cérebro (doutrina cefalocêntrica), abriu as buscas. Os egípcios, há milênios, mantinham um ponto de vista cardiocêntrico. Muito tempo depois, antigos acadêmicos gregos retomaram o tema, mas permaneceram indecisos entre o coração e o cérebro, uma controvérsia que durou séculos. O ponto de vista cefalocêntrico predominou, e seguiu-se uma nova questão, quanto ao lugar dessas funções no cérebro, os ventrículos ou o tecido nervoso, que também durou séculos. Esta última localização, apesar de inicialmente equivocada, prosperou. Entretanto, esta representou apenas um começo, já que mais estudos, nos séculos seguintes, revelaram definições e localizações mais precisas para as funções mentais superiores.

The debates about the soul, mind and its complex higher functions, and attempts to correlate them with a seat in the body, have represented a theme of great interest to innumerable sages (philosophers, physicians, anatomists, thinkers, priests, among others) since ancient times. The search began by questioning which part of the body these functions were located in, the heart or brain.^1^^,^ 2 Here, some historical aspects of the beginnings on the subject, as seen by Western authors, will be outlined.

## THE SEAT OF THE MIND: HEART OR BRAIN

The brain was recognized and named with a specific term (“brain”) for the first time, as registered in the Edwin Smith surgical papyrus, presumably authored by the high-priest and physician Imhotep (ca. 2655-2600 BC). However, the role of the brain was seen as insignificant, being removed and discarded during the mummification procedures, while the heart was regarded as valuable, envisaged as the seat of consciousness and intelligence.^2^^,^ 3 A long hiatus followed, and ancient Greek sages, going back to Homer’s time (ca. 900 years BC), took up the theme. The seat of the soul [mind] divided them - many regarded the heart as the principal organ of the mind (“cardiocentric” doctrine), while others saw the brain as the fundamental organ (“cephalocentric” or “cerebrocentric” doctrine). Both views ran in parallel, a controversy that lasted for many centuries.^1^^,^ 2 The cardiocentric view was advocated first by Empedocles of Agrigentum (495-430 BC), and then by Democritus (460-370 BC) and Aristotle (384-322 BC) [the latter regarded the brain as a mean to cool the heat and simmer the passions of the heart], as well as by Praxagoras of Kos (ca. 340 BC - ?), and Diocles (Medicus) of Carystus (IV century BC).^2^^,^ 4 ^,^ 5 On the other hand, Alcmaeon of Croton (ca. 520-450 BC), Greek physician and anatomist, inaugurated a cephalocentric view, stating that “the governing faculty [*hegemonikon*] is in the brain”, recognizing it as the center of the soul, as well as of the mind, including sensory perception, thought and comprehension.^6^ He influenced prominent figures, such as Pythagoras (582-497 BC), Anaxagoras (500-428 BC), Hippocrates (460 -370 BC), Plato (427-347 BC), Herophilus of Chalcedon (ca. 330-ca. 260 BC), Erasistratus of Chios (ca. 310-ca. 250 BC),^7^ and finally, Galenus of Pergamon (129-ca. 210 AD).^6^^,^ 8

The cephalocentric view ultimately prevailed, enduring until the present day, and the mind and higher functions found their seat in the brain.

It is noteworthy to underscore that the search for a place for the functions was closely accompanied by a humoral doctrine that governed the function of the brain and body, developed by the ancient Greek, and that lasted for a very long period, only declining in the XVIII century (Box).

**Box. The primordial elements, humors and spirits of Greek physiology.**

The ancient Greek authors believed that four “primordial elements” (earth, air, fire, water) and their four “qualities” (heat, cold, wet, dry) (proposed by Empedocles [ca. 500-ca. 430 BC], or earlier),^27^ and “bodily humors” (blood, black bile, yellow bile, phlegm), played a fundamental role in health and disease.^5^^,^ 28

Additionally, there was a “spirit” concept (*pneuma* [Greek], *spiritus* [Latin]), already known since classical antiquity, which represented an essential role in the physiology of the nervous system, and of the entire body.^5^^,^ 10 ^,^ 29 Seemingly, the concept was introduced by Anaximenes of Miletos (ca. 588-ca. 524 BC) (or even earlier, going back at least to Homer), who regarded “air” as the first principle of things, identifying it with life and soul: “As our soul which is air, he says, holds us together, so wind (i.e., breath, pneuma) [spirit] and air encompass the whole world”.^11^^,^ 29 ^,^ 30 Later, Erasistratus begun to elaborate its physiological importance and developed the “spirit doctrine”, describing its formation steps, initially in the liver, the “natural spirit” (*pneuma physikon* or *spiritus naturalis*), from which it reached the heart, and there mixed with the *pneuma* [spirit, breath, air] from the lungs, forming the “vital spirit” (*pneuma zoticon* or *spiritus vitalis*), with access to the entire body, a part reaching the ventricles of the brain to be processed into “animal spirit” or “psychic pneuma” (*pneuma psychikon* or *spiritus animalis*), and stored there.^7^^,^ 9 ^,^ 13 ^,^ 30 Galenus, described the spirit doctrine in a similar way, perfected mainly in the last step, when the vital spirit was transformed into animal spirit after passing the *rete mirabilis* (“wonderful net” or “retiform plexus”), and refined in the choroid plexus. The animal spirit was assumed to flow from the anterior to the other ventricles, and also to permeate the brain substance, underpinning the mechanisms for the functioning of the nervous system and the body as a whole.^5^^,^ 7 ^,^ 10 ^,^ 11

Descartes sustained the spirit doctrine, the animal spirit being produced in the pineal gland, and stored in the ventricles, additionally, the pineal gland was responsible for regulating the flow of the spirit to the meshed part of the brain.^16^

Willis considered that the animal spirit was produced by the cerebral and cerebellar cortical layers, and distributed by the underlying white matter [as previously mentioned by Franciscus Sylvius (1614-1672)], flowing to the *meditullium* [central region], thence to the medulla [brainstem], spinal cord and nerves, and finally to the whole body. He added a new liquid element, the “nervous juice” (*succo nervosum*), a vehicle for the animal spirit (*spirituum animalium vehiculo esse*) for easing its movements, and with nutritious qualities.^15^^,^ 22 ^,^ 24 ^-^ 26

After acknowledgement that the seat of the higher functions was the brain, the cephalocentric view, came the quest to establish in which part of this organ it was located, the ventricles or nervous tissue.

## VENTRICULAR LOCALIZATION

The cephalocentric view evolved further with the discovery of the ventricular system, credited to the pioneering studies of the human brain performed by Herophilus of Chalcedon (ca. 330-ca. 260 BC) and Erasistratus of Chios (ca. 310-ca. 250 BC), Greek physicians and anatomists who revealed novel anatomical structures, and proposed new physiological concepts, during their Alexandrian phase. Herophilus described the ventricles of the brain [apparently he was the first], distinguishing a double anterior and a posterior one, which he saw as the most important, and placed in the latter the soul and the mental functions. His collaborator and follower Erasistratus described the ventricles with three cavities, the double anterior, an intermediate, and a posterior one. He acknowledged the concept of *pneuma* [spirit]) (proposed by Anaximenes in the VI century BC), grouped the spirit into modalities and described their formation [for the first time] (Box).^7^^,^ 9 ^,^ 10

This knowledge was adopted and refined by Claudius Galenus of Pergamon (129-ca. 210 AD), a Greek physician, who recognized a ventricular system with three cavities in communication, with the animal spirit flowing inside. He considered that the basic faculties (perception, cognition, and memory) were located in the cavities [ventricles], while the brain substance was regarded as a kind of template for nervous structure. However, he did not distribute the faculties differentially in these cavities. He accepted the manner of spirit grouping and formation proposed by Erasistratus, and developed it further (Box).^2^^,^ 5 ^,^ 7 ^,^ 10 ^,^ 11

Galenus inherited a solid basis and enhanced the received knowledge. He influenced many other authors that followed, such as Nemesius, the Bishop of Emesa (ca. 350-ca. 420 AD), Poenician [Syrian] theologian and philosopher, who distributed the mind (soul) [mental faculties], together with the animal spirit, among the three known ventricles of the brain (as defined by Erasistratus and Galenus) - sensation (sources of the senses) in the anterior, evaluation in the middle, memory and reminiscence in the posterior.^2^^,^ 12 He appears to be the first to assign the faculties distinctively to each of the three ventricles.^11^^,^ 13

**Figure 1:**
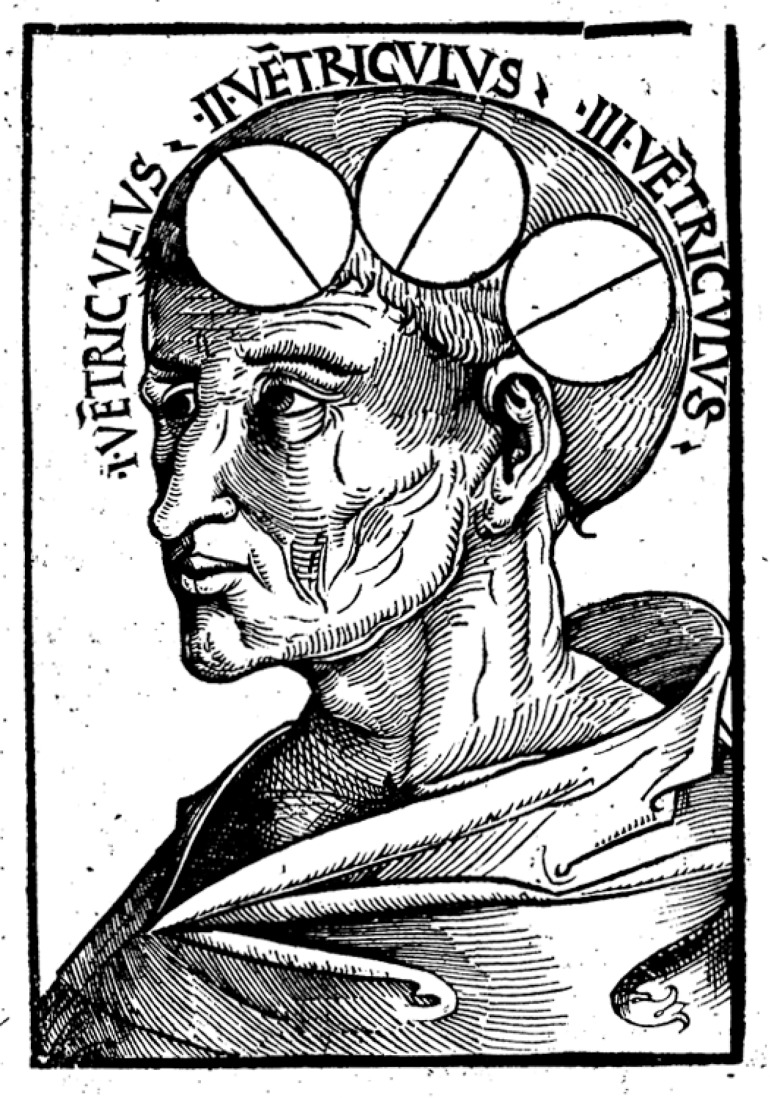
The ventricles from Philosophia Naturalis of Albertus Magnus (1506).^14^ (I) common sense, and basic imagination, (II) creative imagination, phantasy, rational thought, and evaluation, and (III) memory and reminiscence.

A large number of variants of localization patterns followed in the centuries that followed. The most influential appeared to be that of Albertus (Magnus or the Great) (1193-1280), a German bishop and philosopher, later canonized, who described the ventricular system (*concavitate cerebri*) [ventricles] (with flowing animal spirit inside) (Figure 1), where the virtues (*virtute*) [faculties] were allocated - (I) common sense (*sensus communis*) (convergence of the external senses), and basic imagination (*imaginatio*), (II) creative imagination (*imaginativa*), phantasy, rational thought (*cogitatio*), and evaluation (*estimatio*), and (III) memory and reminiscence. An illustration with the three brain cavities was provided in his *Philosophia Naturalis* (1506). Albertus was strongly influenced by Avicenna’s [the Persian physician Ibn-Sina (980-1037)] conceptual views on the issue.^1^^,^ 14 ^,^ 15

The ventricular doctrine, mainly based on the ideas of Albertus, maintained its influence during the remaining Middle Ages, until the Renaissance.

**Figure 2:**
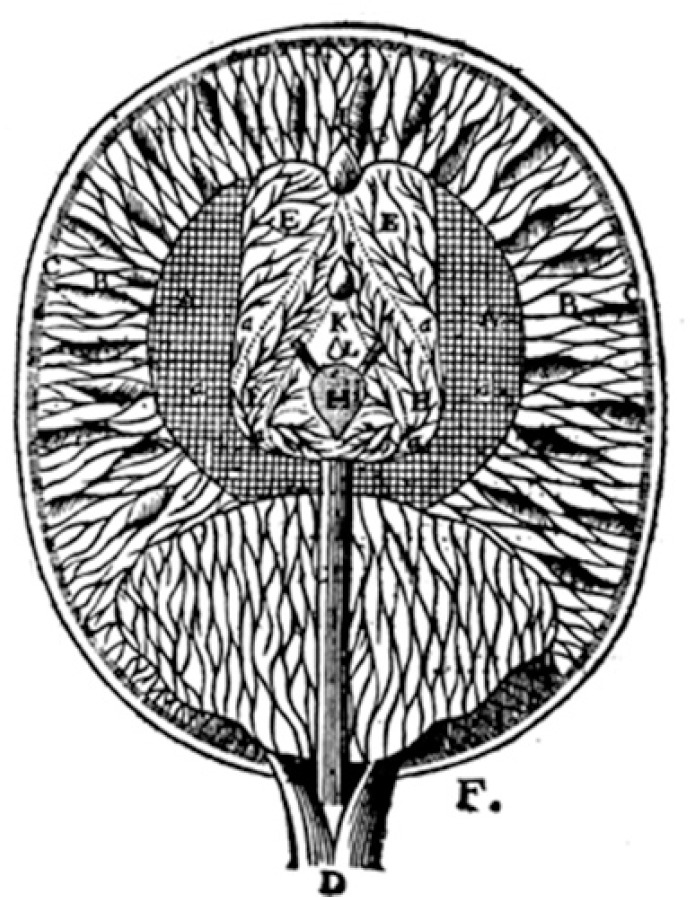
The brain from L’Homme (Tractatus de Homine) of René Descartes (drawings created by Louis de la Forge) ([1633] 1664).^16^ A+B = solid parts of the brain: A (internal part) = net with meshes of small tubes (a) [senses and memory]; B (external part) = twisted filaments projecting from the net, with (c) external attachment ; E = cavities (cavitez) [ventricles]; H = pineal gland [imagination, common sense and “rational soul”].

An alternative understanding to what was already known on the theme was proposed by René Descartes (1596-1650), a French philosopher, physicist, and mathematician. He regarded the ventricles as only one cavity, and the width [solid part] of the cerebrum and cerebellum fashioned by an internal net with meshes of small tubes, and an external part composed by delicate twisted filaments, with intervals (pores) between them. The latter could allow the passage of the animal spirit contained in the ventricles and derived from the pineal gland, the flow being regulated by movements of the latter (Figure 2). The filaments could change their shape, and the pores could be variably enlarged or narrowed according to the force of the inflowing [animal] spirit, a mechanism that constituted the functional basis for the faculties (e.g., formation of a memory trace). He placed some faculties, senses and memory, in the internal part of the brain, and imagination and common sense, as well as the “rational soul”, in the pineal gland ([1633] 1664).^16^^-^ 18

It must be kept in mind that many of Descartes’ basic anatomical and physiological assumptions were peculiar, as seen in light of what was already known in his time.^18^ The ideas of Descartes, with the interaction of the animal spirit with the nervous tissue, regulated by the pineal gland, can be seen as a transitional period of the localization ideas.^16^^,^ 19 ^,^ 20

## NEURAL LOCALIZATION

The ventricular idea began to lose its influence with the systematic use of dissections and autopsies with clinical-pathological correlations.^19^^,^ 20 Vesalius and Willis made important new anatomical and conceptual contributions that changed the former views entirely.

Andreas Vesalius (Andries van Wesel) (1514-1564), a Flemish physician and anatomist, denied that the seat of the mental functions was contained in the ventricles, but stopped short of giving an opinion about the location of the higher functions, citing the limitations of anatomy to explain this issue (1543).^1^^,^ 21

**Figure 3:**
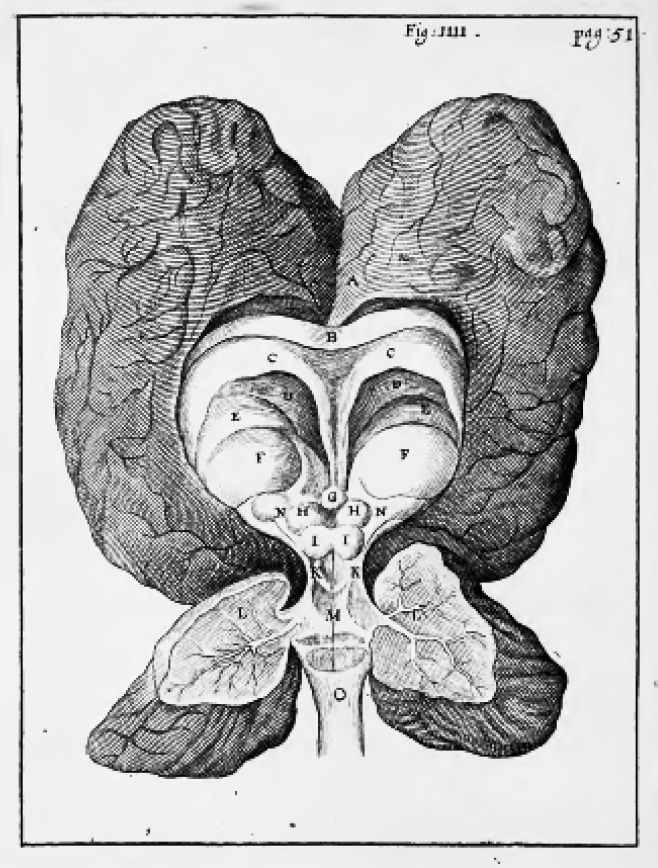
Human brain from Cerebri Anatome of Thomas Willis (drawing by Christopher Wren) (1664).^24^ A = cerebral cortex [memory]; B = corpus callosum [imagination]; D = internal cavities of the brain [serous liquid]; E = tips of the limbs (crura) of the medulla oblongata or striate bodies; F = thalamus (thalami nervorum opticorum); G = pineal gland; H+I = rounded prominences (nates and testes) [instincts]; M+O = medulla oblongata + L = cerebellum [sensation and movements [voluntary] [passions and instincts] [involuntary movements].

He was followed by Thomas Willis (1621-1675), an English physician, who regarded the brain as the primary seat of the “rational soul” (*anima rationalis*) (in man), the origin and source of all movements and concepts, especially imagination, memory, and appetite. He conjectured that memory and remembrance were related to the outer surface of the brain [gyri and circumvolutions], perception and imagination to the corpus callosum, movements [voluntary] and sensation (senses) to the striate bodies (and the medulla oblongata [brainstem]) (although dependent to some extent on the brain). Passions and instincts were related to the “rounded prominences” (*nates* and *testes*) [quadrigeminal bodies], and to the medulla oblongata and cerebellum (the latter also responsible for involuntary movements) (Figure 3). All mentioned activities were dependent on the movements of the animal spirit throughout the tissue, with the help of the “nervous juice”, a concept he introduced (Box). The ventricles, according to him, were filled with water (*aqua*) (in the deceased) (serous liquid), produced by the choroid plexus, beside the presence of waste liquids from the cerebral substance (1664, [1672]1683). He repeatedly cited Cartesius [Descartes], Pierre Gassendi (1592-1655), and others.^15^^,^ 22 ^-^ 25

Thus, Willis specified different solid structures of the brain as the seat of mental faculties. However, he recognized that these assignments were made on a conjectural basis. Thus, despite the inaccuracies, the question was settled in this initial approach.^26^

Finally, after millennia of philosophical thinking, research and speculations, the mind and the higher functions found their place in the brain tissue, the ventricles being relieved from this function. Further studies proceeded, gradually revealing new and more precise definitions and sites for these functions, a quest that would occupy researchers for the centuries to come.

## References

[1] Finger S (1994). Origins of Neuroscience: A History of Explorations into Brain Function.

[2] Rose FC (2009). Cerebral Localization in Antiquity. J Hist Neurosci.

[3] Breasted JH (1930). The Edwin Smith Surgical papyrus (facsimile and hieroglyphic transliteration with translation and commentary, in two volumes).

[4] Gross CG (1995). Aristotle on the brain. Neuroscientist.

[5] Karenberg A (2015). Blood, Phlegm and Spirits: Galen on Stroke. Hist Med.

[6] Celesia GG (2012). Alcmaeon of Croton's observations on health, brain, mind, and soul. J Hist Neurosci.

[7] Turliuc D, Costea CF, Dumitrescu GF, Cucu A, Carauleanu A, Buzduga C, Turliuc S (2015). The protoparents of the cerebral ventricles. Romanian J Artistic Creativity.

[8] Smith W (1873). Erasistratus. A Dictionary of Greek and Roman biography and mythology.

[9] Dobson JF (1927). Erasistratus. Proc Royal Soc Medicine.

[10] Rocca J (1997). Galen and the Ventricular System. J Hist Neurosci.

[11] Manzoni T (1998). The cerebral ventricles, the animal spirits and the dawn of brain localization of function. Arch Ital Biol.

[12] Nemesius (1636). THE Nature Of Man.

[13] Wickens AP (2014). A History of the Brain: From Stone Age surgery to modern neuroscience.

[14] Magnus (1506). Philosophia Naturalis. Mich. Furter.

[15] Swanson LW (2007). Quest for the basic plan of nervous system circuitry. Brain Res Rev.

[16] Descartes R (1664). L'homme et un traitté de la formation du foetus du mesme autheur.

[17] Donaldson IM (2009). The Treatise of man (De homine) by René Descartes. J R Coll Physicians Edinb.

[18] Lokhorst G-J, Zalta Edward N (2017). Descartes and the Pineal Gland. The Stanford Encyclopedia of Philosophy.

[19] Feinberg TE, Farah MJ, Feinberg TE, Farah MJ (1997). The Development of Modern Behavioral Neurology and Neuropsychology. Behavioral Neurology and Neuropsychology.

[20] Toledo-Pereyra LH (2015). Medical Renaissance. J Invest Surg.

[21] Vesalius Andreas (1543). De Humani Corporis Fabrica.

[22] Eadie MJ (2003). A pathology of the animal spirits - the clinical neurology of Thomas Willis (1621-1675). Part I - Background, and disorders of intrinsically normal animal spirits. J Clin Neurosci.

[23] Molnar Z (2004). Thomas Willis (1621-1675), the founder of clinical neuroscience. Nat Rev Neurosc.

[24] Willis T (1664). Cerebri anatome, cui accessit nervorum descriptio et usus. Tho. Roycroft.

[25] Willis T, Pordage S (1683). Two discourses concerning the soul of brutes which is that of the vital and sensitive of man.

[26] Meyer A, Hierons R (1965). On Thomas Willis's Concepts of Neurophysiology. Med Hist.

[27] Singer C (1922). Greek Biology and Greek Medicine.

[28] Jackson WA (2001). A short guide to humoral medicine. Trends Pharmacol Sci.

[29] Fairbanks A (1898). Anaximenes. The First Philosophers of Greece.

[30] Richardson LD (2018). Academic Theories of Generation in the Renaissance.

